# Effects of Previous Land-Use on Plant Species Composition and Diversity in Mediterranean Forests

**DOI:** 10.1371/journal.pone.0139031

**Published:** 2015-09-23

**Authors:** Yacine Kouba, Felipe Martínez-García, Ángel de Frutos, Concepción L. Alados

**Affiliations:** 1 Instituto Pirenaico de Ecología (CSIC), Zaragoza, Spain; 2 Departamento de Sistemas y Recursos Naturales, Escuela de Ingeniería de Montes, Forestal y del Medio Natural, Universidad Politécnica de Madrid, Madrid, Spain; 3 Instituto Pirenaico de Ecología (CSIC), Jaca, Huesca, Spain; Technical University in Zvolen, SLOVAKIA

## Abstract

At some point in their history, most forests in the Mediterranean Basin have been subjected to intensive management or converted to agriculture land. Knowing how forest plant communities recovered after the abandonment of forest-management or agricultural practices (including livestock grazing) provides a basis for investigating how previous land management have affected plant species diversity and composition in forest ecosystems. Our study investigated the consequences of historical “land management” practices on present-day Mediterranean forests by comparing species assemblages and the diversity of (i) all plant species and (ii) each ecological group defined by species’ habitat preferences and successional status (i.e., early-, mid-, and late-successional species). We compared forest stands that differed both in land-use history and in successional stage. In addition, we evaluated the value of those stands for biodiversity conservation. The study revealed significant compositional differentiation among stands that was due to among-stand variations in the diversity (namely, species richness and evenness) of early-, intermediate-, and late-successional species. Historical land management has led to an increase in compositional divergences among forest stands and the loss of late-successional forest species.

## Introduction

Most of today’s unmanaged Mediterranean forests were once under intensive management [1–3]. Since antiquity, those forests have been extensively cleared, and the rate of forest loss accelerated in the 18th and 19th C. [4]. In Euro-Mediterranean countries, in particular, many of the natural forests were coppiced for timber and firewood, which created coppices that differed in their management intensity and the time since coppicing had ceased.

On the other hand, changes in socioeconomics and production systems in the late 19th C. and early 20th C. resulted in the abandonment and subsequent afforestation (both spontaneously and through planting) of the poorest arable lands and many pastures [5–7]. In many regions, abandonment and forest encroachment occurred in several phases, which created a complex mix of forest stands that are at different stages of succession [8–10]. In Spain, as in many other European countries, the largest increase in forest cover on abandoned lands that had been used for agriculture or as pastures occurred in the second half of the 20th C. [11].

In the early 21st C., those human-altered forests have continued to bear the imprint of historical changes in land-use [9]. In particular, the type of prior land-use and intensity have had a strong influence on the characteristics of secondary forest stands [12,13], and the type of historical management has had a similarly strong effect on abandoned coppice stands [14,15]. Furthermore, historical logging, livestock grazing, and agriculture might have affected the diversity and composition of the plant communities in those forests [8,9,16]. The plant community differs depending on the time since human disturbance has ceased. Classifying the plant species (e.g. early-, intermediate-, and late-successional species) found within the successional stages can increase our understanding of the successional dynamics [8,17]. Investigating how plant species diversity and composition differ among forest stands that have different land-use histories and are at different successional stages can increase our understanding of the consequences of past land-use on plant communities in these forests and provides a basis for predicting the responses of the communities to future disturbances and environmental changes. Furthermore, given the increase in human-altered forests in the Mediterranean Basin [1], a greater understanding of the contribution that these forests can make to biodiversity conservation is crucial [18].

Plant diversity and compositional differences between primarily old growth forests and secondary growth forests [6,10,19,20] and between managed and unmanaged forests [4,21,22] have been well studied; however, published studies of formerly managed forests and secondary growth forests in Mediterranean environments are rare. Studies have emphasized the importance of anthropogenic disturbances in fostering the establishment of early-successional species to the disadvantage of forest specialists [23]. A recent review [14] found that a comparison between two types of forest management (even-aged vs. uneven-aged) did not provide a clear pattern that could predict the plant diversity response; however, other studies have shown that uneven-aged stands have the capacity to maintain high plant diversity [15].

Our study was conducted in oak forests that included formerly managed stands and secondary forest stands that differed in their structural properties. The main objective was to investigate the effects of previous forest management and agricultural activities (including livestock grazing) on contemporary forest plant communities. Specifically, we first assessed the effects of stand history (abandoned coppices vs. secondary growth stands), stand age (young vs. old), age structure of the forest stand (even-aged vs. uneven-aged stands), and the extent of the canopy cover on plant species composition. Secondly, we assessed whether those factors affected the species diversity (namely, species richness and evenness) of each ecological group defined by species’ habitat preferences and successional status (i.e. early-, intermediate-, and late-successional species). Previous studies have suggested that diversity as a measure of community response can be deceptive if there are increases or decreases in the number of species in the plant groups; e.g., increases in the abundances of early-successional species might obscure the loss of or decrease in late-successional species [15,24]. Thirdly, we evaluated the value of the oak stands for biodiversity conservation as reflected by their ability to support species that are associated with natural forests (i.e., late-successional species), which can be achieved using indicator species analysis [25,26] that allows the assessment of the strength of the relationship between species abundance and forest type. Previous studies have shown that the potential to develop into forests that are more natural is high in forests that have high diversity in late-successional species [27,28]. We hypothesized that differences in historical land-use have led to the development of forest stands that have contrasting successional trajectories, which increases the floristic differentiation among oak stands [18]. Furthermore, previous land management has reduced the diversity of forest specialists in those oak stands [1,18].

## Materials and Methods

### Ethics statement

All plant surveys were performed under permits issued by the municipal councils of Nueno and Arguis, and by the Director of the Parque Natural de la Sierra y Cañones de Guara, Aragón, Spain.

### Study area

**Fig 1:**
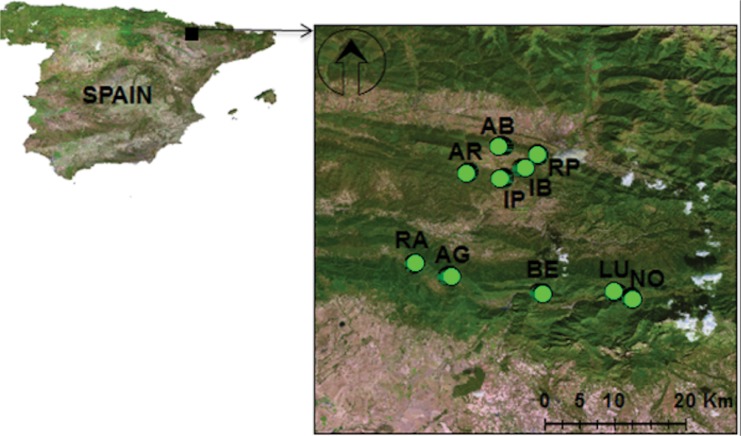
Study area. The location of the study area in Spain (left panel), and the locations of the stands that were surveyed in the Central Pre-Pyrenees (right panel) (AB Abena, AG Arguis, AR Ara, BE Belsué, IB Ibort, IP Ipies, LU Lucera, NO Nocito, RA Rasal, RP Rapun).

The study was carried out in oak forests at a moderate elevation (800–1000 m a.s.l.) in the Central Pre-Pyrenees, Spain (between 42.32 N and 42.11 N, and 0.31 W and 0.04 W) (Fig 1). Most of the lithology is conglomerate, limestone, marl, and sandstone developed on Eocene flysch sedimentary formations. The climate is transitional sub-Mediterranean; i.e., influenced by continental effects from the north and by milder Mediterranean conditions from the south (i.e., the Ebro Basin). Mean annual precipitation is 1317 ± 302 mm (1915–2005) and mean annual temperature is 11.5 ± 2.8°C (1910–2005) [29].

The oak forests (mainly, *Quercus faginea*) of the Central Pre-Pyrenees are a mosaic of stands that differ in their structure and history. Based on historical land-use, those semi-deciduous oak forests are of two types: abandoned coppice stands (coppices that differed in historical coppicing intensities and time since management abandonment) and secondary growth stands (most of which were established on abandoned farmlands and pastures, primarily, in the second half of the 20th C. [30]). The overstorey canopy of those semi-deciduous oak stands was mostly *Q*. *faginea* interspersed with some scattered pines (*Pinus sylvestris* and *Pinus nigra*) and evergreen oak (*Quercus ilex* subsp. *ballota*). The understory comprised shrubs (mainly, *Quercus coccifera*, *Buxus sempervirens*, *Genista scorpius*, *Juniperus communis*), forbs (mainly, *Aphyllanthes monspeliensis*, *Arenaria montana*, *Achillea millefolium*), and graminoids (mainly, *Brachypodium pinnatum*, *Carex halleriana*, *Festuca rubra*, *Carex flacca*, *Bromus erectus*).

### Data collection

Based on historical land-use maps and aerial photographs from 1957 and 2006, we selected ten *Q*. *faginea*-dominated stands that were at different successional stages and had different land-use histories [29,30] (Table 1). In 2009 and 2010, during the period of peak growth (May and June), vascular plant species were surveyed in the ten stands. Within each stand, three 500-m linear transects (N = 30 transects) were established. To estimate plant abundance, richness, and species composition within each transect, we used the Point-Intercept Method [31], which involves recording, at 40-cm intervals, the identity of all individuals that are in contact with a vertical nail [32].

**Table 1:** Characteristics of the ten *Quercus faginea* stands surveyed in the Central Pre-Pyrenees, Spain.

Abbreviation	Location	FORTYPE	AGE (year)	CVAGE (%)	CANCOV (%)
AB	Abena	SF	Old (50)	EA (19)	47.7, 49.3, 48.4
AR	Ara	CS	Young (35)	EA (17)	39.8, 29.3, 29.6
AG	Arguis	CS	Old (50)	EA (10)	38.9, 47.1, 45.4
BE	Belsué	CS	Young (40)	UEA (43)	43.9, 47.2, 41.0
IB	Ibort	CS	Old (63)	EA (17)	75.4, 68.6, 63.6
IP	Ipies	CS	Old (64)	EA (15)	08.3, 15.9, 39.9
LU	Lucera	CS	Young (39)	EA (12)	53.5, 45.6, 60.9
NO	Nocito	SF	Old (56)	UEA (47)	50.4, 36.6, 44.4
RP	Rapun	CS	Old (69)	EA (9)	44.6, 46.6, 49.6
RA	Rasal	SF	Young (31)	UEA (31)	46.0, 41.2, 29.3

FORTYPE: forest type; AGE: mean stand tree age; CVAGE: coefficient of variation of stand age; CANCOV: canopy cover; SF: secondary growth stand; CS: abandoned coppice stand; EA: even-aged stand; UEA: uneven-aged stand. CANCOV is reported for the three transects of each stand.

We recorded all of the vascular plants that touched the nail and any overstorey species (including *Q*. *faginea*) that was above the nail (S1 Appendix). Plant species that could not be identified with certainty in the field were collected, pressed, and brought to the laboratory for identification by botanists. Species that have traits that make them difficult to distinguish were identified to genus, only. Plant nomenclature followed *Flora Iberica* [33]. The abundance of each plant species in each transect was calculated as the number of points where the species occurred. In each transect, canopy cover (CANCOV) was estimated based on the relative abundance (%) of woody species (including trees and large shrubs) that were ≥ 1.5 m tall. For each stand, we estimated the age of ~ 40 trees (for details on age estimations see [29]), calculated mean tree-age and the coefficient of variation of tree-age. Two binary variables were derived from the age data: AGE (young vs. old stands) and CVAGE (even-aged vs. uneven-aged stands). Forest type (FORTYPE; secondary growth stands vs. abandoned coppice stands) of each stand was based on observations in the field (see Table 1).

### Grouping species by successional status

Analyses based on ecological groups can help to identify the mechanisms that underlie the tree species-plant diversity relationship [34]. In our study, species were clustered within one of three ecological groups based on species’ habitat preferences and successional status [23,35]: Early-successional species (ES), which are shade-intolerant pioneer species that reach maximum abundance in open-canopy and disturbed areas, Intermediate-successional species (IS), which occupy young to mature, open- or closed-canopy forests, but not excessively disturbed habitats, and late-successional species (LS), which are shade tolerant species that reach maximum abundance in mature, closed-canopy, forest interiors (S1 Appendix). To assign each plant species to one of the three ecological groups, we used information in the literature [33,36–40], experience in the field, and personal knowledge (Dr. Felipe Martínez-García, botanist).

### Statistical analyses

To quantify the effects of the explanatory variables (AGE, CVAGE, FORTYPE, and CANCOV) on species composition, we used a permutational multivariate analysis of variance (PERMANOVA), which is a multivariate, nonparametric analogue of the univariate analysis of variance (MANOVA). The species abundance data (all species included) were subjected to PERMANOVA using the Bray-Curtis distance measure and 10000 permutations. To identify patterns in the compositional variation of plant communities among stand types (i.e., young vs. old, even-aged vs. uneven-aged, secondary growth vs. abandoned coppice stands), we used unconstrained ordination, non-metrical multidimensional scaling (NMDS). In addition, to test for the effect of canopy cover, this variable was plotted as a smooth surface in an ordination diagram.

Differences in species richness and species evenness among stand types (i.e., young vs. old, even-aged vs. uneven-aged, secondary growth vs. abandoned coppice stands) and the relationship between these two measures of diversity and canopy cover were analyzed using ANCOVA, both for all species combined and for each ecological group. To account for spatial dependencies, stand location was included as a random factor in the ANCOVA models. Normality and homogeneity of variance were tested by examining the model residuals versus the fitted plots and the normal *q-q* plots of the models. The appropriate transformations were used as required.

Indicator species analyses were used to assess the strength of the correlations between plant species and the following forest classes that resulted from the interaction AGE × CVAGE × FORTYP: old even-aged secondary growth stands, old even-aged coppice stands, old uneven-aged secondary growth stands, young even-aged coppice stands, young uneven-aged secondary growth stands, and young uneven-aged coppice stands. Indicator species in each forest class were identified by calculating the indicator values [25] based on plant species abundance (all species included), then we assigned each selected species to an ecological group (i.e., early-, intermediate-, or late-successional species).

The statistical analyses were performed using the programming language R [41]. The PERMANOVA and the NMDS were performed using the R package ‘*vegan*’ [42], the ANCOVA was performed using the R package ‘*nlme*’ [43], and the Indicator Species Analysis was performed using the R package ‘*indicspecies*’ [44].

## Results

In the ten oak stands in the Central Pre-Pyrenees, we identified 206 vascular plant species (S1 Appendix). The most abundant species, which comprised 60% of all of the individuals recorded, were *Buxus sempervirens* (19.17%), *Brachypodium pinnatum* (13.38%), *Aphyllanthes monspeliensis* (8.93%), *Carex halleriana* (5.95%, IS), *Genista scorpius* (4.50%), *Carex flacca* (4.10%, IS), and *Festuca rubra* (3.61%).

The classification of plant species by successional status indicated that most (56%) of the 206 vascular plant species were ES species. Twenty-six percent and 18% of the plants were IS species and LS species, respectively. *Genista scorpius* (ES), *Teucrium chamaedrys* (ES), *Thymus vulgaris* (ES), *Buxus sempervirens* (IS), *Festuca rubra* (IS), *Amelanchier ovalis* (IS), *Aphyllanthes monspeliensis* (IS), *Brachypodium pinnatum* (IS), and *Rubia peregrina* (LS) were the most commonly occurring species (found in all transects) (S1 Appendix).

### Factors affecting species composition

The PERMANOVA analysis revealed that plant species composition differed significantly between stand types (i.e., young vs. old, even-aged vs. uneven-aged, secondary growth vs. abandoned coppice stands). In addition, canopy cover “CANCOV” and plant species composition were significantly correlated.

**Fig 2:**
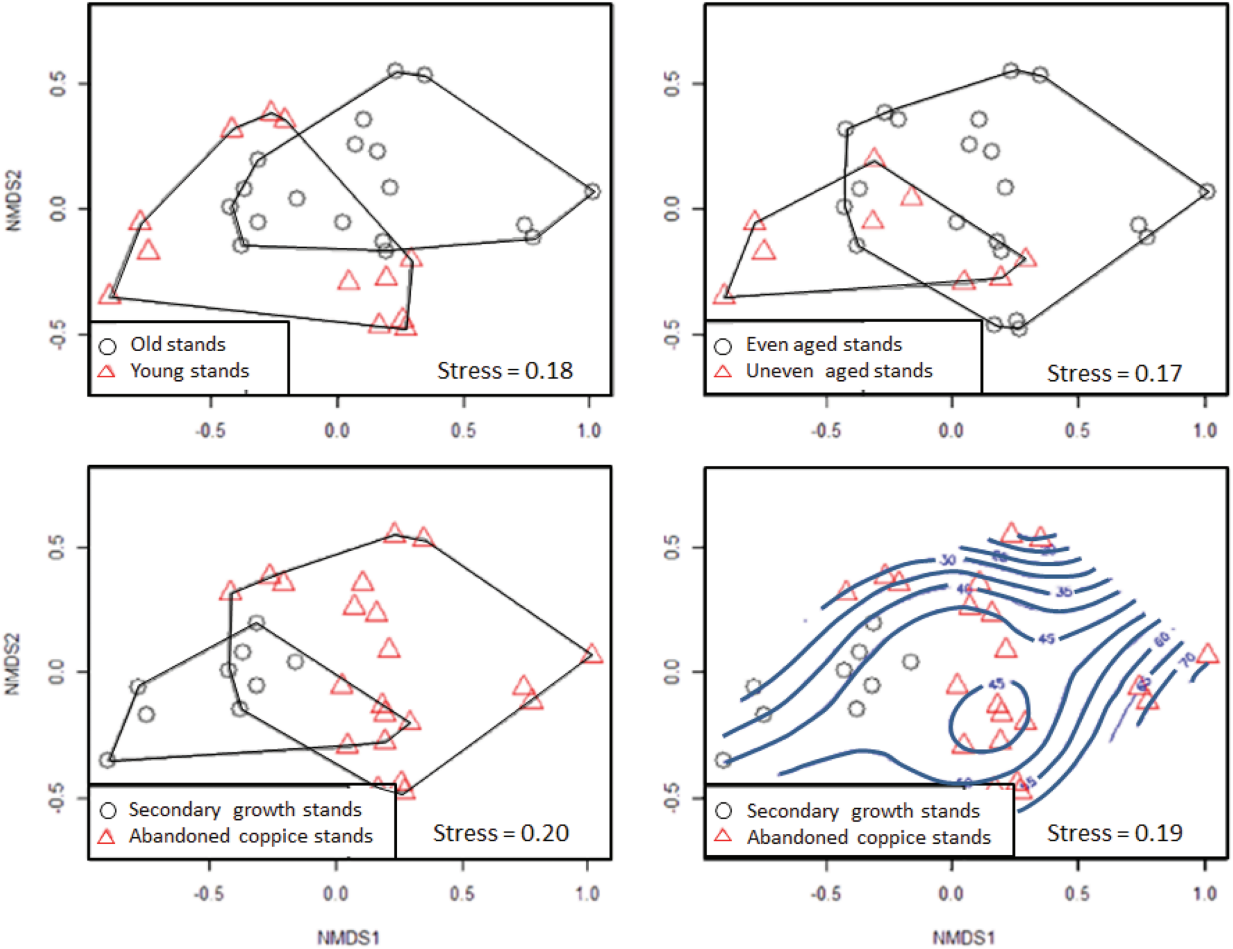
Nonparametric multidimensional scaling (NMDS) ordinations. NMDS diagrams indicating the groupings of the floristic transects by stand type of *Quercus faginea* in the Central Pre-Pyrenees, Spain: young vs. old stands, even aged vs. uneven aged stands, secondary growth stands vs. abandoned coppice stands. Canopy cover is indicated in the ordination diagram as a smooth surface (below right hand plot).

The explanatory variables explained almost 40% of the variation in the composition of plant communities (Table 2). “FORTYPE” explained the largest proportion (15.2%) of the compositional variation, followed by “CANCOV”, which explained 11% of the variation. “AGE” and “CVAGE” each explained ~ 7% of the variation. Fig 2 shows the grouping of plant transects by stand type in the ordination space determined by the NMDS analysis.

**Table 2:** Non-parametric MANOVA test for the effects of explanatory variables on the plant species composition of ten *Quercus faginea* forest stands in the Central Pre-Pyrenees, Spain.

	*F*	*R* ^*2*^	*P*
AGE	2.6	0.072	<0.001
CVAGE	2.6	0.071	0.004
FORTYPE	3.8	0.152	<0.001
CANCOV	7.6	0.110	<0.001

AGE: Stand Age; CVAGE: coefficient of variation of stand age; FORTYPE: forest type; CANCOV: Canopy Cover. R^2^ is the variance (%) explained by each variable.

### Factors affecting the species diversity of each successional species group

Each of the four explanatory variables explained a significant amount of the variation in overall plant species richness and, collectively, they explained >70% of the total variance (Table 3). Based on the variance explained by each variable, “FORTYP” was the most important variable, followed by “CANCOV”, “AGE”, and “CVAGE” (Table 3).

**Table 3:** ANCOVA Test for the effects of four explanatory variables on plant species diversity (species richness and evenness) in ten oak stands in the Central Pre-Pyrenees, Spain.

		Richness			Evenness		
		*R* ^*2*^	*F*	*P*	*R* ^*2*^	*F*	*P*
AS	AGE	**0.150**	**12.8**	**<0.001**	-	0.08	0.770
	CVAGE	**0.107**	**9.2**	**0.006**	-	0.01	0.980
	FORTYPE	**0.291**	**25.0**	**<0.001**	-	0.00	0.459
	CANCOV	**0.160**	**13.7**	**<0.001**	**0.324**	**11.61**	**0.004**
ES	AGE	**0.150**	**16.04**	**<0.001**	-	2.68	0.123
	CVAGE	**0.146**	**15.60**	**<0.001**	-	3.25	0.091
	FORTYPE	**0.326**	**34.72**	**<0.001**	-	0.00	0.985
	CANCOV	**0.156**	**16.98**	**<0.001**	**0.291**	**11.88**	**0.003**
IS	AGE	-	0.17	0.904		0.09	0.764
	CVAGE	**0.122**	**5.35**	**0.029**		0.01	0.981
	FORTYPE	**0.197**	**8.61**	**0.007**		1.04	0.316
	CANCOV	**0.150**	**6.53**	**0.017**	**0.324**	**10.11**	**0.002**
LS	AGE	-	0.4	0.538	-	0.97	0.340
	CVAGE	-	0.0	0.962	**0.293**	**10.2**	**0.003**
	FORTYPE	-	1.6	0.219	-	1.03	0.322
	CANCOV	-	0.7	0.412	-	0.21	0.648

AS: all plant species; ES: Early-successional species; IS: Intermediate-successional species; LS: Late-successional species. Significant effects (P < 0.05) are shown in bold. R^2^ is the variance (%) explained by each significant explanatory variable.

**Fig 3:**
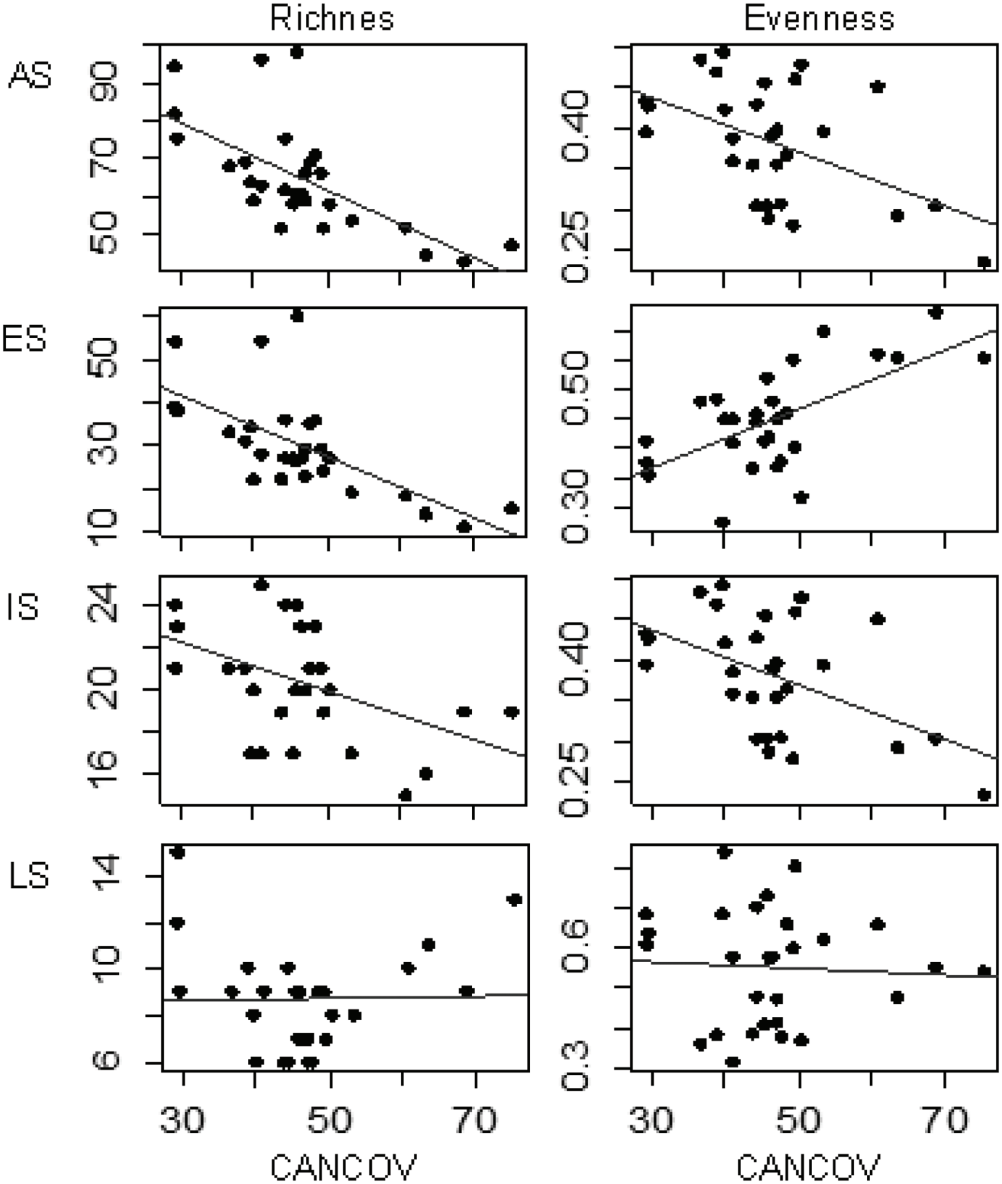
Influence of predictor variables on plant species diversity in ten oak stands in the Central Pre-Pyrenees, Spain. Species richness and evenness (AS: all plant species; ES: Early-successional species; IS: Intermediate-successional species; LS: Late-successional species) as influenced by stand age “AGE” (O: old stands; Y: young stands), age structure of stand “CVAGE” (EA: even-aged stands; UEA: Uneven-aged stands), and forest type “FORTYPE” (SF: secondary growth stands; CS: abandoned coppice stands). Boxes that have the same letter did not differ significantly based on ANCOVA.

**Fig 4:**
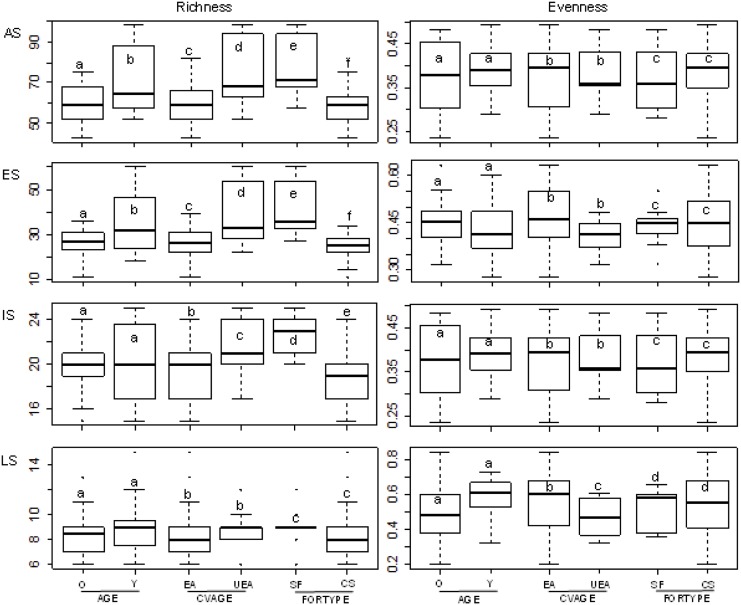
Effects of canopy cover on plant diversity in ten oak stands in the Central Pre-Pyrenees, Spain. Canopy cover (CANCOV) and species richness and evenness (AS: all plant species; ES: Early-successional species; IS: Intermediate-successional species; LS: Late-successional species).

Secondary growth stands that had been established on abandoned lands, young stands, and uneven-aged stands had the highest plant species richness (Fig 3), which was negatively correlated with “CANCOV” (Fig 4). Using successional species groups, the same trends in species richness were apparent among ES and IS species (Table 3; Figs 3 and 4), although the effect of “AGE” among IS species was not statistically significant (Table 3). None of the explanatory variables had a significant effect on the species richness among LS species (Table 3). Among ES and IS species, evenness was affected by “CANCOV” and, among LS species, “CVAGE” had a significant effect on species evenness (Table 3). Species evenness among LS species was significantly higher in even-aged stands than it was in uneven-aged stands (Fig 3). Furthermore, evenness was positively correlated with “CANCOV” among ES species, but was negatively correlated with “CANCOV” among IS species (Fig 4).

### Indicator species

Overall, 70 species were significant (*p* < 0.05) indicator species, and most (39 species) were indicative of young uneven-aged coppice stands, followed by young uneven-aged secondary growth stands (12), old even-aged secondary growth stands (7), old uneven-aged secondary growth stands (7), and young even-aged coppice stands (4) (Table 4, S2 Appendix). One significant indicator species was found in old even-aged coppice stands. LS indicator species occurred in all of the forest classes except old even-aged secondary growth- and coppice-stands; however, young uneven-aged coppice stands harbored the highest number of LS indicator species (Table 4).

**Table 4:** The number of early- (ES), intermediate- (IS), and late-successional (LS) Indicator Species identified in each forest class found in the ten *Quercus faginea* forest stands surveyed in the Central Pre-Pyrenees, Spain.

Forest class	ES	IS	LS	Total
Old even-aged secondary growth stands	2	5	0	7
Old even-aged coppice stands	1	0	0	1
Old uneven-aged secondary growth stands	3	2	2	7
Young uneven-aged secondary growth stands	3	8	1	12
Young even-aged coppice stands	1	2	1	4
Young uneven aged coppice stands	32	4	3	39

Indicator species are those that had significant (*p < 0*.*05*) indicator values in the multilevel pattern analysis.

## Discussion

Our study has demonstrated the importance of previous land-use and forest management in shaping the development of plant species assemblages and the richness and evenness of plant species. Evidently, human-induced disturbances can have a strong influence on plant communities in forest ecosystems, which have been reported elsewhere [8,9,19].

### Plant species composition

The oak stands differed significantly in species composition, which underscores the importance of considering compositional differences in addition to differences in diversity parameters [45]. The evenness of all species collectively did not differ significantly among stand types, which indicated that the differences in species composition were not because of differences in species abundances; rather, they were mainly due to differences in species richness. Furthermore, the high overall richness in young stands (vs. old stands), secondary growth stands (vs. abandoned coppice stands), and uneven-aged stands (vs. even-aged stands) suggest that the compositional dissimilarities were not due to species turnover, only, but were the result of species turnover and nestedness [46]. That is, part of the compositional differentiation is due to species replacement and another part is due to differences in richness between stands; i.e., the richest stands have species that are not present in the poorest stands [46].

### Plant species diversity

Overall species richness was significantly higher in secondary growth stands than it was in abandoned coppice stands, which might have been because of the high number of ES and IS species in the secondary growth stands, which drove the dissimilarities that were apparent in plant community composition between secondary growth stands and abandoned coppice stands (see above). In stands that developed on abandoned agricultural terraces, the persistence of old-field species might have contributed to the high total richness in these stands [8]. The secondary growth stands in our study area were established on formerly abandoned lands (i.e., agricultural terraces and pastures) that were on relatively flat land, and some were in valley bottoms where the soils have large amounts of nutrients and water [29,47]. Those conditions strongly favor shade-intolerant, ruderal, and competitive species [4], which can increase total richness.

The large number of ES colonizers was responsible for the high species richness in young oak stands, which might have contributed to the differences in the plant species composition of young and old stands (see above). ES species are the first to colonize disrupted or damaged ecosystems [9,48]. In our study, the disappearance (i.e., competitive exclusion) of ES species in the transition from one successional stage to another might have been responsible for the comparatively low overall species richness in old (> 50 yr) stands. The richness of ES and IS species was higher in uneven-aged stands than it was in even-aged stands, which might explain the dissimilarities in the plant community composition of even-aged and uneven-aged stands (see above). Those dissimilarities might have occurred because of the high resource availability caused by the formation of gaps in the canopy of uneven-aged stands [14], which can provide conditions that favor the establishment of ES and IS species [49]. In contrast, in even-aged stands, the structure of the overstorey canopy, the amount of interspecific competition, and the resources available on the forest floor change more dramatically and phases of development occur more uniformly than they do in uneven-aged stands, which might reduce the richness of shade-intolerant species [17], most of which are ES and IS species.

Overall species richness and the species richness of ES and IS species were negatively correlated with the extent of the canopy cover. Many studies have demonstrated the negative effect of canopy closure on shade-intolerant species [50,51]. The reduction in the availability of light as a forest canopy closes can reduce species richness and limit the growth and survival of many species that became established during the stand initiation stage, and allow LS species to persist, only [50,52]. Furthermore, the high abundance of the competitor shrub *Buxus sempervirens* in stands that have closed canopies might out-compete other shade-intolerant species by reducing the amount of resources (i.e., light, soil nutrients, water) in the understorey (the abundance of this competitor species was positively associated with the extent of the canopy cover). In our study, the explanatory variables did not explain a significant amount of the variation in the richness of LS species, which indicates that the number of LS species did not differ significantly between stand types. Similarly, [23] found that forest stands that differed in management intensity did not differ in the number of LS species. In the oak forests in the Central Pre-Pyrenees, the extent of canopy cover and ES species evenness were positively correlated, perhaps, because canopy closure increases interspecific competition, which can lead to the exclusion of rare species and increase the mortality rate of highly abundant ES species. Probably, the reduction in sunlight caused by canopy closure contributed to a reduction in the evenness of IS species [4]. Furthermore, that reduction might have been due to the greater dominance of the competitor shrub *B*. *sempervirens* in stands that had closed canopies.

The evenness of LS species was highest in even-aged stands, which have a more homogeneous structure and are more uniformly limiting in shade and microhabitats than are uneven-aged stands [14,53]. Under those conditions, interspecific competition inhibits the establishment of shade-intolerant species, but favors the establishment of LS species that can thrive in those environments [50,54–56], which can increase the abundance of uncommon LS species and, thereby, increase species evenness. Other studies have suggested that facilitation or release from inhibition by preceding successional species, or intrinsic characteristics such as arrival time, growth rate, and the absence of direct interaction with early species might lead to the establishment of LS species [12].

### Indicator species

Indicator species analysis revealed that, in the absence of old uneven-aged coppice stands in our study area, young uneven-aged coppice stands have a high value for biodiversity conservation. In addition to harboring the highest number of significant indicator species, those stands included a sizeable portion the LS indicator species (e.g., *Brachypodium sylvaticum*, *Lathyrus linifolius*, and *Ranunculus repens*). We predict that LS species will progressively replace the ES and IS indicator species (e.g., *Brachypodium distachyon*, *Plantago lanceolata*, *Dactylis glomerata*) identified in our study. The chronic fluctuations in resources that occur in the successional process might lead to the reordering of plant species: species loss and colonization by species that are better suited to the new environment (in this case, LS species) [57]. The uneven-aged secondary growth stands, only, harbored significant LS indicator species, and the highest number were in old uneven-aged secondary growth stands (i.e., *Cornus sanguinea*, *Rubia peregrine*). Variation in the ages of trees in uneven aged stands increases the variety of habitats, which permits the coexistence of species that have different requirements [14,15]; however, the ability of those stands to provide habitat for LS species makes them very important for biodiversity conservation.

### Implications for biodiversity conservation

Agriculture, livestock grazing, and forest coppicing, which were once common types of land management in the Mediterranean Basin, were abandoned in many places in the late 19th C. and 20th C. After the abandonment of marginal agricultural lands, pastures, and coppices, forest stands that differed in land-use histories and structural properties developed. The coexistence of different types of stands can provide heterogeneous habitats that maximize biodiversity conservation at the regional scale [18]. Furthermore, although our study focused on the presence of plant species in previously disturbed oak forests, we recognize that these forests provide vital resources for fauna, ecosystem services, and forest products [58]. Therefore, the conservation of those oak stands might provide ecosystem and economic benefits (e.g., ecotourism) and should be promoted through government incentives and land-use regulations. The uneven-aged oak stands and, specifically, the abandoned uneven-aged coppice stands seem to represent a progression in the transition towards native forests, which increases their importance from a conservation perspective.

## Supporting Information

S1 Appendix Plant species abundance. Plant species abundance (mean ± SD) in the 10 oak forests surveyed in the Central Pre-Pyrenees, Spain. Successional status: ES, Early-successional species; IS, Intermediate-successional species; LS, Late-successional species.
(XLSX)

S2 Appendix Multilevel pattern analysis. A test of the relationship between plant species and forest class in the 10 oak forests surveyed in the Central Pre-Pyrenees, Spain.
(DOCX)
