# Supplementary material for: Effects of Previous Land-Use on Plant Species Composition and Diversity in Mediterranean Forests
Source: PLoS One. 2015 Sep 23;10(9):e0139031. doi: 10.1371/journal.pone.0139031 (PMC4580598; doi:10.1371/journal.pone.0139031)
Supplement: S2 Appendix — A test of the relationship between plant species and forest class in the 10 oak forests surveyed in the Central Pre-Pyrenees, Spain. (DOCX) [file pone.0139031.s002.docx]

**S2 Appendix. Multilevel pattern analysis.** A test of the relationship between plant species and forest class in the 10 oak forests surveyed in the Central Pre-Pyrenees, Spain.

| Forest class/ indicator species | Indicator value | p-vales | Successional status |
| --- | --- | --- | --- |
| Old even-aged secondary growth stands |  |  |  |
| *Rhamnus saxatilis* | 0.818 | 0.018 | IS |
| *Berberis vulgaris* | 0.816 | 0.028 | IS |
| *Thymus praecox* | 0.811 | 0.013 | IS |
| *Polygala calcarea* | 0.764 | 0.023 | ES |
| *Linum narbonense* | 0.724 | 0.04 | IS |
| *Dorycnium pentaphyllum* | 0.585 | 0.024 | IS |
| Old even-aged coppice stands |  |  |  |
| *Lathyrus filiformis* | 0.764 | 0.02 | ES |
| Old uneven-aged secondary growth stands |  |  |  |
| *Viburnum lantana* | 0.951 | 0.001 | IS |
| *Globularia nudicaulis* | 0.874 | 0.015 | ES |
| *Polygala alpestris* | 0.805 | 0.004 | ES |
| *Carex flacca* | 0.8 | 0.018 | IS |
| *Cornus sanguinea* | 0.786 | 0.024 | LS |
| *Rubia peregrina* | 0.748 | 0.001 | LS |
| *Cheirolophus intybaceus* | 0.73 | 0.05 | ES |
| Young even-aged coppice stands |  |  |  |
| *Echinospartum horridum* | 0.988 | 0.001 | IS |
| *Paronychia kapela* | 0.816 | 0.006 | ES |
| *Hieracium murorum* | 0.73 | 0.013 | LS |
| *Rosa sp.* | 0.646 | 0.039 | IS |
| Young uneven-aged secondary growth stands |  |  |  |
| *Rhamnus alaternus* | 1 | 0.001 | IS |
| *Stipa eriocaulis* | 0.894 | 0.003 | ES |
| *Juniperus phoenicea* | 0.886 | 0.004 | IS |
| *Lithodora fruticosa* | 0.819 | 0.018 | ES |
| *Bupleurum ranunculoides* | 0.813 | 0.008 | ES |
| *Cytisophyllum sessilifolium* | 0.805 | 0.002 | LS |
| *Quercus coccifera* | 0.793 | 0.013 | IS |
| *Thalictrum alpinum* | 0.774 | 0.001 | IS |
| *Teucrium pyrenaicum* | 0.743 | 0.032 | IS |
| *Trinia glauca* | 0.707 | 0.032 | IS |
| *Amelanchier ovalis* | 0.7 | 0.001 | IS |
| *Juniperus oxycedrus* | 0.649 | 0.032 | IS |
| Young uneven aged coppice stands |  |  |  |
| *Brachypodium distachyon* | 1 | 0.002 | ES |
| *Coronilla scorpioides* | 1 | 0.002 | ES |
| *Plantago lanceolata* | 1 | 0.002 | ES |
| *Xeranthemum inapertum* | 1 | 0.002 | ES |
| *Medicago sativa* | 0.993 | 0.002 | ES |
| *Thapsia villosa* | 0.969 | 0.001 | ES |
| *Stachys recta* | 0.93 | 0.005 | IS |
| *Prunella laciniata* | 0.918 | 0.007 | IS |
| *Dactylis glomerata* | 0.904 | 0.01 | ES |
| *Arrhenatherum elatius* | 0.878 | 0.01 | ES |
| *Convolvulus cantabrica* | 0.844 | 0.009 | ES |
| *Prunus spinosa* | 0.824 | 0.009 | IS |
| *Alyssum alyssoides* | 0.816 | 0.031 | ES |
| *Arenaria leptoclados* | 0.816 | 0.031 | ES |
| *Brachypodium sylvaticum* | 0.816 | 0.031 | LS |
| *Centaurea alba* | 0.816 | 0.032 | ES |
| *Crepis vesicaria* | 0.816 | 0.031 | ES |
| *Lathyrus linifolius* | 0.816 | 0.032 | LS |
| *Phleum pratense* | 0.816 | 0.031 | IS |
| *Ranunculus repens* | 0.816 | 0.031 | LS |
| *Sideritis hirsuta* | 0.816 | 0.024 | ES |
| *Veronica orsiniana* | 0.816 | 0.031 | ES |
| *Vicia sativa* | 0.806 | 0.028 | ES |
| *Vicia cracca* | 0.805 | 0.044 | ES |
| *Medicago lupulina* | 0.804 | 0.022 | ES |
| *Potentilla neumanniana* | 0.8 | 0.027 | ES |
| *Melica ciliata* | 0.797 | 0.014 | ES |
| *Eryngium campestre* | 0.789 | 0.015 | ES |
| *Galium verum* | 0.789 | 0.033 | ES |
| *Silene vulgaris* | 0.776 | 0.017 | ES |
| *Hypericum perforatum* | 0.766 | 0.048 | ES |
| *Achillea millefolium* | 0.756 | 0.034 | ES |
| *Santolina chamaecyparissus* | 0.756 | 0.049 | ES |
| *Poa angustifolia* | 0.732 | 0.028 | ES |
| *Helianthemum marifolium* | 0.717 | 0.018 | ES |
| *Galium lucidum* | 0.644 | 0.035 | ES |
| *Hippocrepis ciliata* | 0.642 | 0.021 | ES |
| *Teucrium chamaedrys* | 0.632 | 0.004 | ES |
| *Thymus vulgaris* | 0.611 | 0.006 | ES |

ES, Early-successional species; IS, Intermediate-successional species; LS, Late-successional species. Only species that had a significant indicator value (p < 0.05) are included in the table.
